# Creating an Extremely Long-lasting Neuroischemic Wound Model

**DOI:** 10.1016/j.xjidi.2024.100328

**Published:** 2024-11-16

**Authors:** Sufan Chien, Harshini Sarojini, Arezoo Rajaee, Mohammad Bayat, Samson Chien, Girish Kotwal

**Affiliations:** 1 Department of Surgery, University of Louisville School of Medicine, Louisville, Kentucky, USA

**Keywords:** Animal model, Neuroischemia, Rabbit, Long term, Wound

## Abstract

In wound study and dressing development, a lack of a suitable animal model that can recapitulate the complex pathophysiology of human chronic wounds has been a major hurdle. Chronic wounds are defined as wounds that heal with a significant delay, usually over a period >2–3 months, but no current animal wound model has such a longischemia. After a longexploration, our group has developed an animal wound model with ischemia and nerve damage lasting for at least 6 months. This model can be easily combined with other conditions such as diabetes and aging for wound mechanistic study and critical testing of dressings. This report presents the method that has significant utility in evaluating therapies that could become the future standard for screening all new wound dressings.

## Introduction

Management of chronic wounds has become a major global therapeutic challenge that is expected to escalate with increasing conditions that impede wound healing (eg, aging, diabetes, obesity, and vascular disorders) (Izadi and Ganchi, 2005; Nunan et al, 2014). More than 3000 wound dressings have been developed in the past century (Dhivya et al, 2015), but the best available treatment achieves only a 50% healing rate, which is still temporary (Wu et al, 2007).

There are many reasons for these failures, but a lack of a suitable animal model that sufficiently recapitulates the features of human chronic wounds is an issue that has challenged scientists and clinicians for decades. The statement that “Old ulcers in 1830 will be the older ulcer in 1860” made in one of the first papers describing chronic wounds (Hamilton, 1854) still applies today. With no breakthrough, old ulcers in 2024 will be older ulcers in 2054.

Over 100 known physiologic factors contribute to wound-healing deficiencies in individuals with diabetes (Brem and Tomic-Canic, 2007), but one fundamental clinical observation is that wounds do not heal in tissue that does not bleed, whereas they always heal in tissue that bleeds profusely (Gottrup, 2004). Problems of hypoxia surrounding wound tissues have been known for decades (Allen et al, 1997; Hopf et al, 1997), and once an ulcer develops, the blood supply required for healing is several fold higher than that required for intact skin (Lepantalo et al, 2000). Reduced oxygen tension can also predict the risk of wound infection (Hopf et al, 1997). For many chronic diabetic foot ulcers, ischemia may not be the initiating factor because most ulcers start from a combination of repeated trauma, neuropathy, pressure loading, and/or infection. Nevertheless, ischemia is the main cause that hinders healing, regardless of its initiation (Fang and Galiano, 2008; Medina et al, 2005). For chronic venous insufficiency, ischemia was not considered a factor in the past, but extensive research in recent years has confirmed the ischemic nature of chronic venous insufficiency (Gschwandtner et al, 2001; Moysidis, 2012), which arises from the combination of microvascular dysfunction (Junger et al, 2000), capillary thromboses (Franzeck et al, 1993), reduced capillary density, microvascular tortuosity (capillary tufts), and formation of fibrin cuffs (Gourdin, 1993). The end result is an extremely low transcutaneous oxygen pressure at the venous ulcer rim (Franzeck et al, 1993).

Generating a true long-term ischemic animal wound model has been a long-time challenge ever since the wound study started. Chronic wounds are defined as wounds that heal with a significant delay, usually >2–3 months (Sisco and Mustoe, 2003). In the past, the longest ischemic time was up to 14 days (Gould et al, 2005), but this time was too short. The rabbit ear ischemic model reported 3 decades ago maintained an ischemic time of up to 28 days (Ahn and Mustoe, 1990), and it has been widely used in ischemic wound studies (Ahn and Mustoe, 1990; Wu and Mustoe, 1995). Our group modified the technique with a minimally invasive approach that reduced skin damage and increased the success rate (Chien, 2007; Chien and Wilhelmi, 2012). However, this modification did not increase the ischemic time. Some groups repeat the surgery at days 5, 10, 17, and 21 to disrupt any reconstituted blood flow to maintain longer ischemia (Bonomo et al, 2000), but this is impractical in animals with reduced resistance, such as aging or diabetes. In addition, when these wounds appear in the clinic, the initial stage of development is long past (Dhall et al, 2014). Without an animal model that simulates the environment of chronic wounds, an understanding of the mechanisms that produce this type of wound or delay healing remains out of reach (Salcido et al, 2007). This article reports an improved model of rabbit ear with months even years of ischemic time and discusses some of the wound-healing mechanisms that are coming to light.

## Results

### Ear appearance

**Figure 1:**
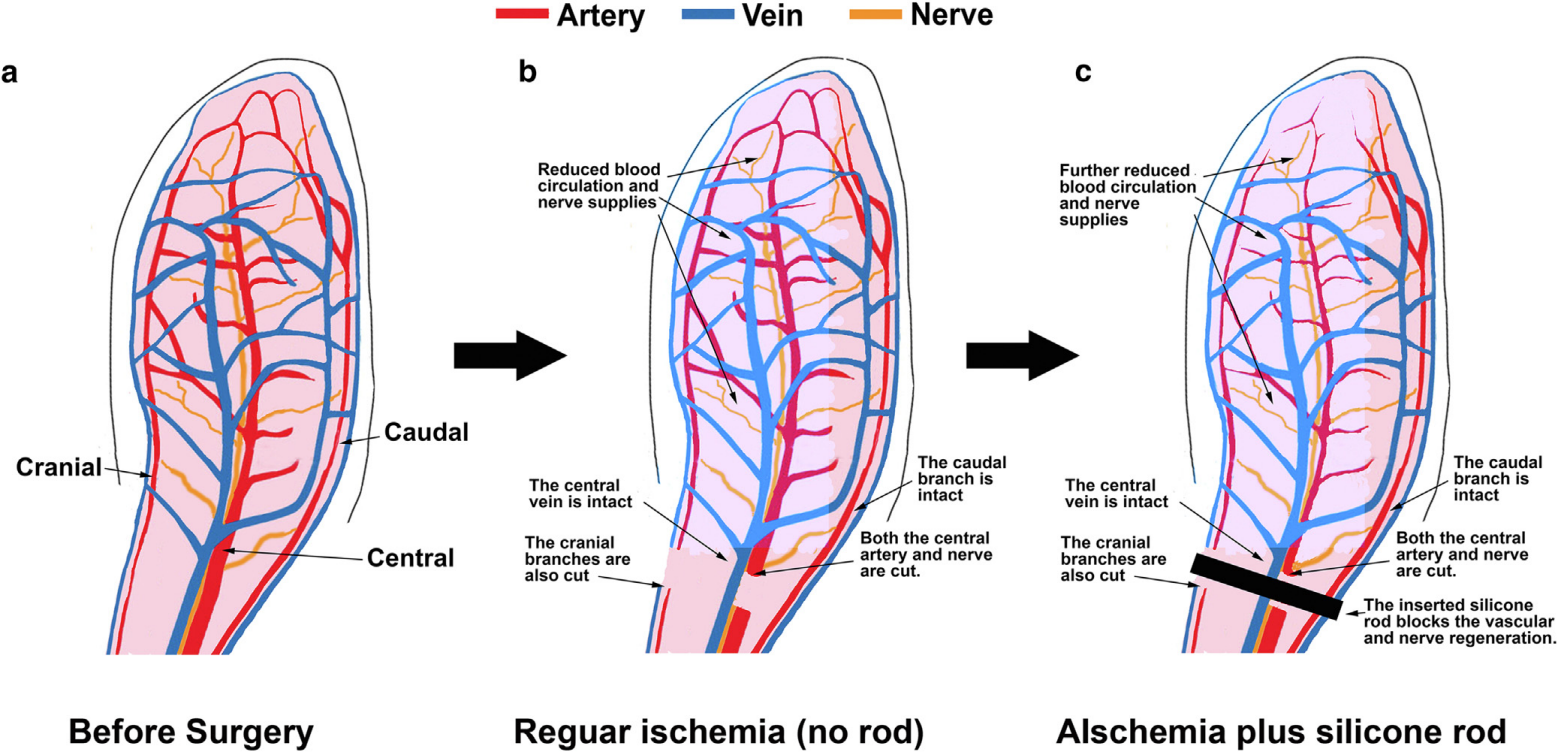
**Rabbit ear and types of ischemic wound models.** Provided is an explanation of ischemic wound models in rabbit ear. **(a)** Before surgery. The ear is perfused by 3 bundles of blood vessels accompanied by nerves—central, cranial, and caudal. The central bundle is the largest to provide the main blood and nerve supply to the ear. **(b)** Regular ischemia (without silicone rod implant). The artery and nerve in the central bundle are cut, leaving only the central vein functional in this bundle. The cranial bundle is totally cut, leaving the caudal branch intact. **(c)** Ischemia with a silicone rod implant. After the regular ischemia is induced, a silicone rod (or O-ring) is inserted into the subcutaneous tunnel to block the vessel and nerve formation, significantly extending the ischemic time. In the regular ischemia without silicone rod implant, the ischemia occurs at the early time but disappears after about 1 month. In the ischemia with a silicone rod implant, the ischemia lasts for more than 6 months.

**Figure 2:**
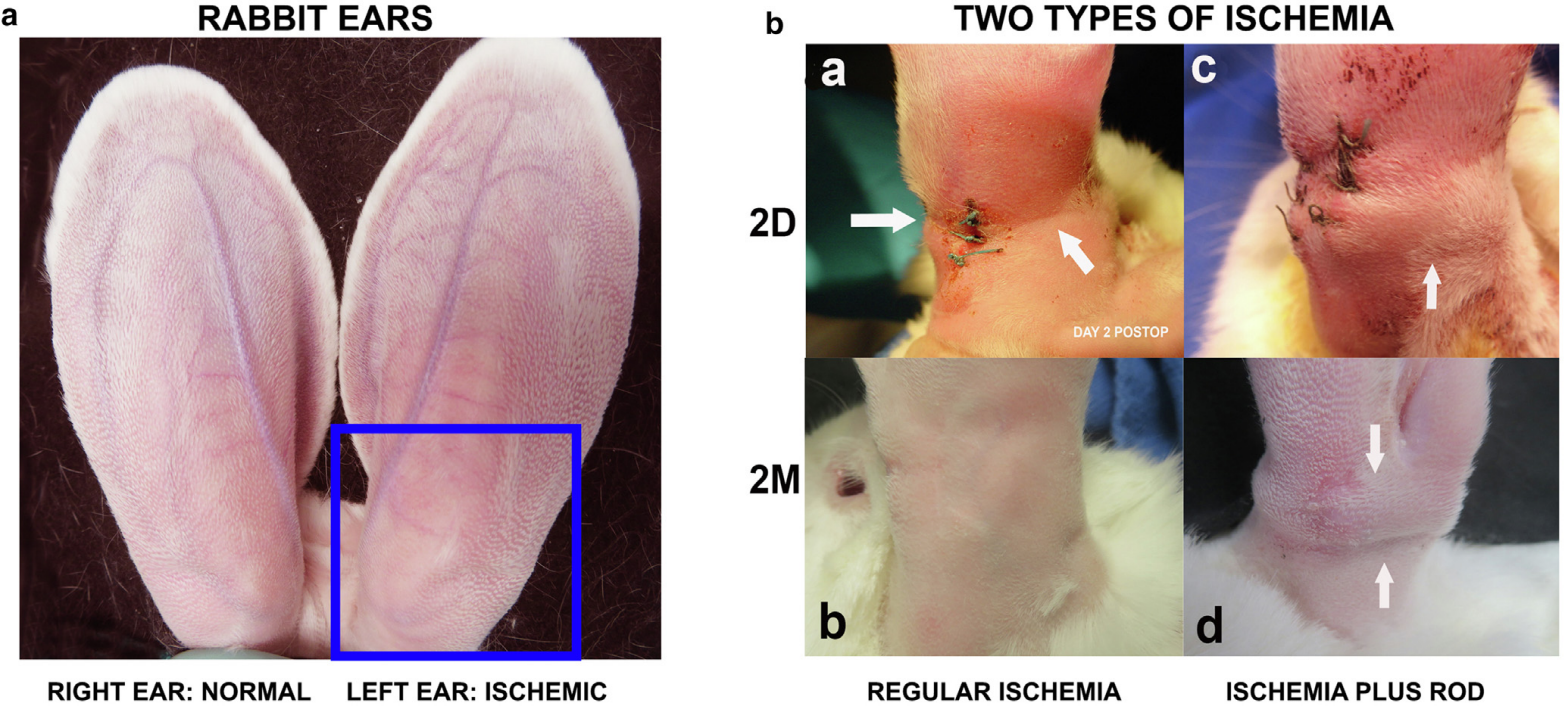
**Overview of the ears and the surgical procedure site.** Outlooking of the ears before and after surgery is shown. **(a)** Normal photo of rabbit ears. In our study, the right ear is always kept normal blood circulation and nerve supply as a control. We create ischemia on the left ear only (the blue square at the ear base). **(b)** Outside comparison of ear base between **(a, b)** regular ischemia and **(c, d)** with silicone rod implant. The silicone rod implant makes the ear base “pregnant” looking **(c, d)** whereas there is no such expansion in regular ischemia **(a, b)** 2 days (top) and 2 months (bottom) after surgery.

The central approach for this study is to generate an ischemic ear that does not become normal for a much longer period of time. In regular ischemia, we reduced ear circulation by cutting the central artery and the whole cranial branch, leaving only the central vein and the whole caudal branch intact (Figure 1A and B). In the extended ischemia, we used a silicone rod (or ring) buried in the ear tunnel to prevent the collateral circulation formation and nerve recovery, resulting in a much longer ischemic time (Figure 1C). The ear in the regular ischemic rabbit had the same appearance as previously reported by our group (Figure 2Ba and b) (Chien, 2007; Chien and Wilhelmi, 2012). The ears of the silicone rod–implanted rabbits showed the “bump” of the implanted silicone rod at the ear base (Figure 2Bc and d).

### Ear perfusion measurements

**Figure 3:**
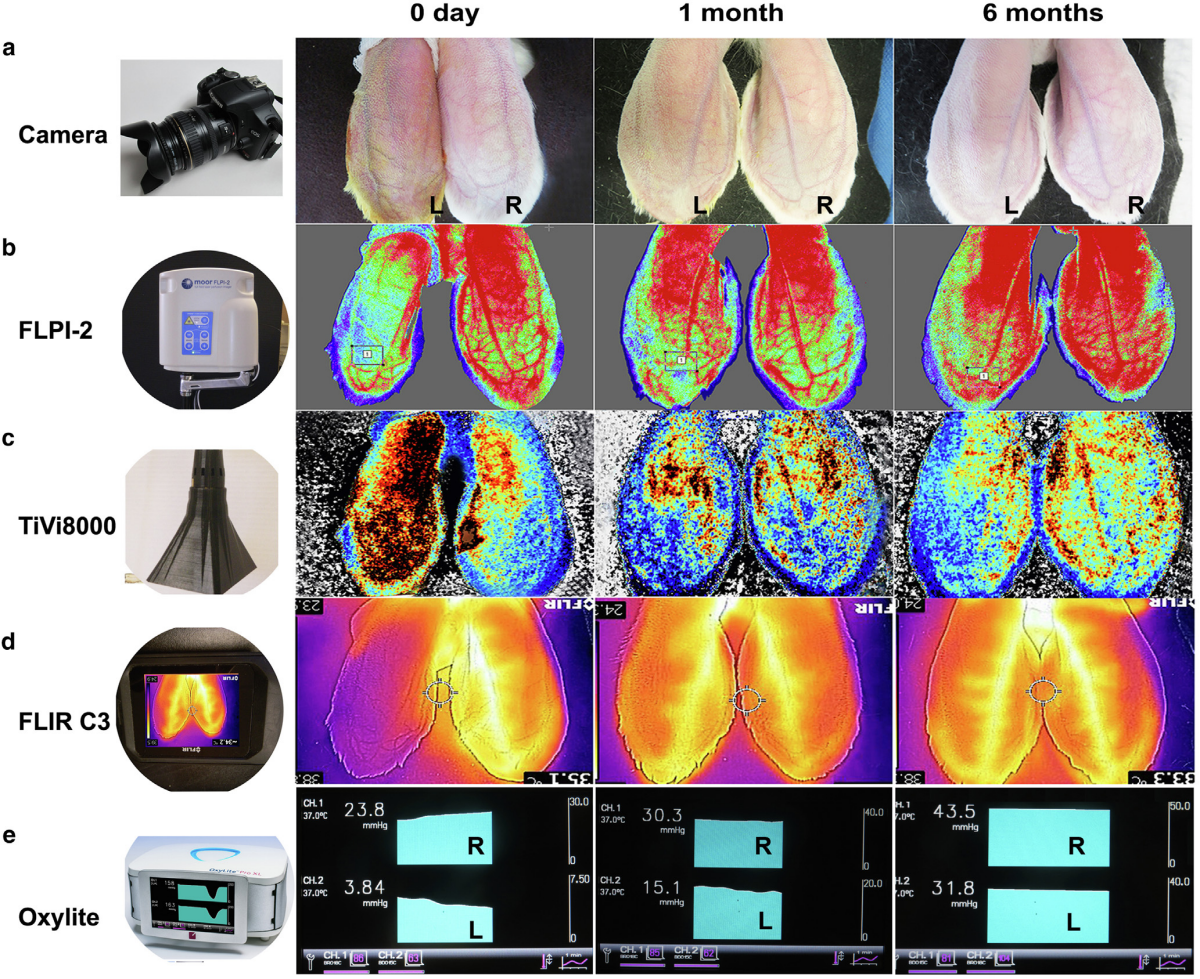
**Measurement equipment.** Shown are rabbit ears at 0 day, 1 month, and 6 month with photos and different pictures using different machines for perfusion studies. **(a)** Regular ear photos taken by a normal camera. A color change can be seen on the left ear at day 0, but the color change is not clearly seen at 1 and 6 months. **(b)** Ear perfusion photos by Moor FLPI-2. This machine can clearly see the perfusion difference between the ischemic (left) and nonischemic (right) ears 0 day, 1 month, and 6 months after surgery. **(c)** Photos shown by TiVi8000. This machine can also see the difference between the ischemic and nonischemic ears at 0 day, 1 month, and 6 months, but the differences are not as clear as compared with that of Moor FLPI-2 owing to its lower resolution. **(d)** Photos shown by FLIR C3 camera. Similar to TiVi8000, we can see the perfusion differences between the ischemic and nonischemic ears, but the results are not clear as those of the Moor FLPI-2 owing to its lower resolution. **(e)** Photos shown by Oxylite Pro. Right ear (ch. 1): normal; left ear (ch. 2): ischemic. This machine can clearly show the temperature differences between the 2 ears at 0 day, 1 month, and 6 months. The only disadvantage is that it requires a subcutaneous probe placement to get the correct temperature readings. In panels **a–d**, the left ears are ischemic, whereas the right ears are nonischemic. In panel **e**, the top channels are normal, whereas the bottom channels are ischemic.

We used multiple techniques for noninvasive or minimally invasive detection of ear tissue microvascular perfusion (Figure 3). This includes regular light camera (Figure 3a), Moor FLPI-2 laser speckle contrast imager (Moor Instruments), which is currently the standard imaging system for microvascular perfusion measurements (Figure 3b). We also have a Wheels Bridge’s TiVi8000 micro camera for the evaluation of red blood cell concentration in the skin (Figure 3c). For skin temperature measurements, we have an infrared thermometer (Thermo Fisher Scientific), and a new FLIR C3 infrared thermal imaging system (Global Test Supply), which can map skin temperature with region of interest for exact spot temperature measurements (Figure 3d). For subcutaneous oxygen pressure, oxygen saturation, and carbon dioxide tension measurements, we have an OxyLite Pro oxygen tension monitor (Oxford Optronix) (Figure 3e), Moor VMS-OXY (Moor Instruments). Some of them are presented briefly below.

#### Laser speckle Doppler imaging (Moor FLPI-2)

The FLPI-2 instrument has been the preferred equipment for noninvasive microvascular perfusion measurements (FLUX). The newer version has much better stability and is less affected by ambient light. For most rabbits, FLPI-2 measurements indicated extremely reduced perfusion immediately after surgery. The moorFLPI-2 blood flow imager uses the laser speckle contrast technique to deliver real-time, high-resolution blood flow images, providing very good performance in a wide range of preclinical and clinical research applications. It has optical zoom to assess small areas right up to a full-size adult hand with a single imager. The moor FLPI2 features an ergonomically designed scan head and highly refined software package, which promotes a smooth workflow and enables the high throughput required to scan cohorts quickly with good accuracy. In our study, perfusion recovered gradually in both the regular and the silicone rod–implanted ischemic groups, but reduced perfusion could persist for many months longer in the silicone rod–implanted ischemic group (Figure 3b).

#### Tissue viability determination by micro camera (TiVi8000)

We also used the Wheels Bridge TiVi8000 Tissue Viability Imaging system (Linköping) for perfusion measurements. It provided a good image if the skin did not have color changes. However, if the skin had artificial color markers (such as ink or betadine residue) or edema, it could give incorrect results with increased perfusion (Figure 3c). Owing to its relatively lower resolution, the accuracy is not as good as that of moorFLPI-2.

#### Thermal imaging (FLIR C3)

This old technique has found new life because of recent technological advancements (Hernandez-Contreras et al, 2016). We used the FLIR C3 camera, which showed temperature distributions and comparisons between different ears (Figure 3d). The C3 has a relatively lower resolution, but their multiple newer models can provide improved resolution and ranges.

### Skin temperature

Skin temperature was measured with an infrared thermometer (Thermo Fisher Scientific) to compare the normal and ischemic ears. The temperature difference between the normal ear and the ischemic ear was increased immediately after surgery, but it reduced with time. By 1 month, the difference had essentially disappeared in most rabbits in the regular ischemic group. In the silicone rod–implanted group, the temperature difference showed similar trends, but the temperature difference from normal ear existed for more than 6 months (Figure 3d and e), which fits the results of FLUX difference measured by Moor FLPI-2.

### Subcutaneous oxygen saturation and/or oxygen tension

We have used several machines to measure transcutaneous oxygen tension, oxygen saturation, and carbon dioxide tension, including the Radiometer Tina TCM-4 (Radiometer) (Grouiller et al, 2006), Bionet Vet BM5 (Bionet), and Moor VMS-OXY (Moor Instruments) instruments. A normal transcutaneous oxygen tension is above 40 mmHg, whereas a reading below 20 mmHg indicates ischemia.

The Tina TCM-4 required a longer time to obtain a correct perfusion reading. The Moor VMS-OXY worked better but still had a problem with stability. The most accurate equipment for oxygen tension measurement was the OxyLitePro (Oxford Optronix), which gave exact oxygen pressure readings but required probe needle placement in a precise tissue space (Figure 3e).

### Extremely long-time ischemia and delayed wound healing

#### More than 6 months of ischemia with silicone rod implant

**Figure 4:**
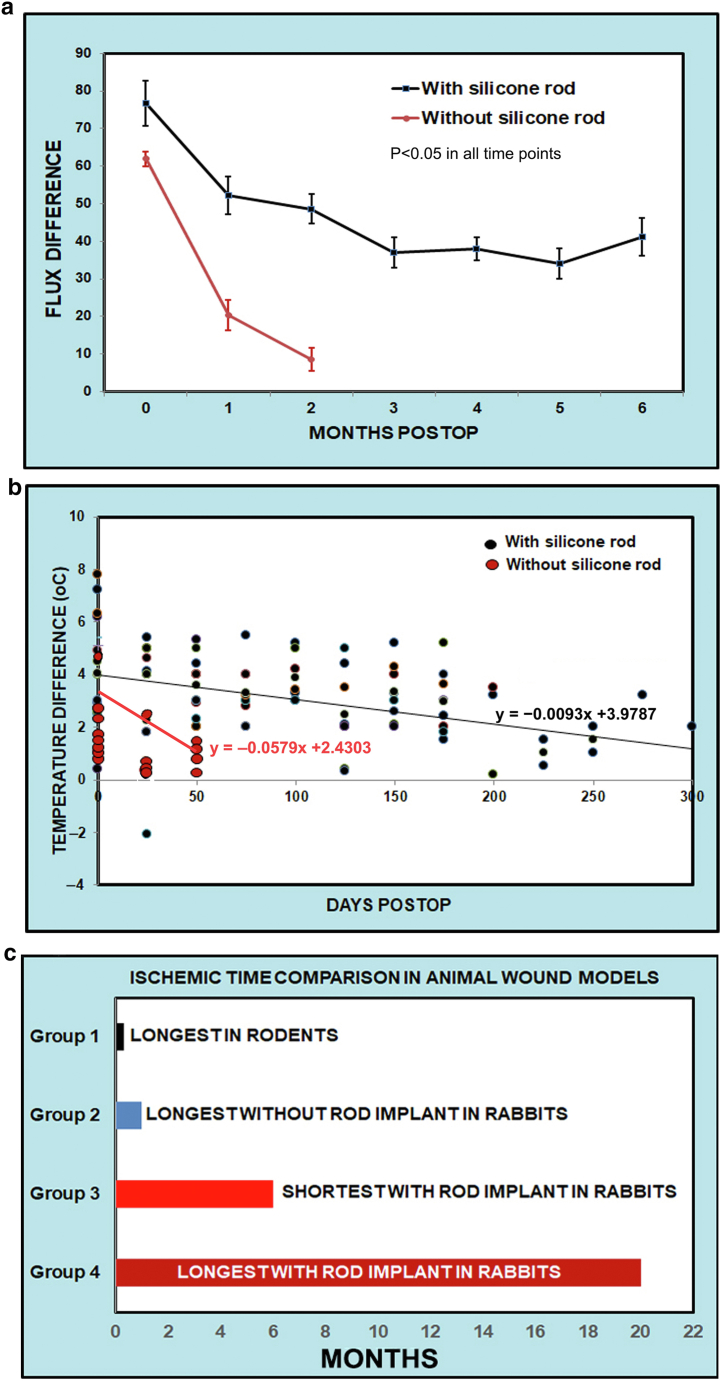
**Ear perfusion differences.** Comparison of ischemic times in different approaches and differences in skin perfusion in the rabbit ear ischemic model is presented. **(a)** Red blood cell Flux difference between ears in the 14 animals kept for 6 months with and without silicone rod implants. Ear tissue perfusion differences were clearly shown by the Flux measured monthly with the FLPI-2. For silicone rod–implanted rabbits (black line), the Flux difference between the rod-implant and no-rod-implant ears (the same rabbit) was 76.8 ± 9.2 (mean ± SEM, *P* < .05). In the no-silicone rod-implant rabbits (red line), the difference at day 0 was 61.9 ± 3.1 (mean ± SEM) but decreased quickly after 1 month and almost disappear after 2 months. Please note that these values might not be obtained at the exact the same hour after surgery; even a few hours, the differences can result in big change in the same group. **(b)** Ear temperature differences between normal and ischemic ears. Black: with silicone implanted, the temperature difference between the normal ears and ischemic ears continued at 6 months after surgery (n = 40). Red: in regular ischemic ears (without silicone rod implant), the temperature differences between ischemic ears and normal ears were significant at the beginning but decreased quickly, especially after day 35 (n = 23). Some rabbits were measured more than once a day owing to ear temperature changes rapidly. **(c)** Different ischemic times in history and the currently available animal models used for ischemic wound research. Group 1: historically used the longest ischemic time in rodent skin ischemic models, which could keep the ischemic time up to 14 days (Gould et al, 2005). Group 2: a major increase of ischemic time up to almost 1 month for wound research using rabbit ear ischemic model (Ahn and Mustoe, 1990; Chien, 2007). Group 3: in this study, with the silicone rod implanted in the rabbit ear, ischemic difference between the ischemic and nonischemic ears is clearly seen 6 months after surgery when they were killed (n = 14). In group 4, we intentionally kept 4 animals up to 20 months, but the difference between their ischemic and nonischemic ears were still clearly seen. Because groups 1 and 2 are from the representative literature, they do not have any comparison, we could not add ±SEM to them. The animals in the group 3 and group 4 were killed artificially at 6 and 20 months. However, the differences between groups were still clearly seen. For this reason, we do not place error bars for these timelines—we do not want to put artificial sacrificed time as natural.

The ischemic time in the silicone rod–implanted group extended far beyond our original expectations, and 6 months is the “normal” ischemic time in this group. Ear tissue perfusions clearly shown by the Flux differences with and without silicone rod implant were measured monthly with the FLPI-2. Right and left ears were analyzed and compared in the 14 animals kept for 6 months with silicone rods (treated) and without silicone rods (nontreated). The mean ± SEM of the difference in FLUX between the treated and untreated ears (the same rabbit) was significantly greater in silicone rod–treated ears than in those without silicone rods (*P* < .05, 1-way ANOVA) (Figure 4a). We also compared the same group at different time periods, and we can also clearly see the changes during the months after surgery.

To show the significance of this study, we checked the literature to see what the best ischemic time previous models could achieve. The historically longest ischemic time in rodent skin models was up to 14 days (Gould et al, 2005). A major increase of ischemic time up to almost 1 month was a rabbit ear ischemic model developed in 1990s and thereafter (Ahn and Mustoe, 1990; Chien, 2007). In this study, the silicone rod implanted in the rabbit ear increased the ischemic time to at least 6 months. However, we intentionally kept 4 animals up to 20 months or longer, but the difference between their ischemic and nonischemic ears were still clearly seen from FLUX difference (Figure 4b); because the no-silicone group had the FLUX value <10, we only presented them to 2 months.

Owing to the high cost of upkeep, these animals were killed at 6 months while the differences were still clearly seen. Figure 4c is a summary of ischemic time comparison among animal models so far. Groups 1 and 2 are the longest ischemic times from history until recently (Ahn and Mustoe, 1990; Gould et al, 2005). Group 3 is our limited number (14) of rabbits that were kept for 6 months, and group 4 has only 4 rabbits that were kept for 20 months, with 1 intentionally kept up to 28 months and still exhibited a perfusion difference compared with normal ears. In the regular ischemic group, the difference gradually disappeared within 1 month. The silicone rod implantation has changed the dynamics of collateral formation and has produced the longest ischemic time that, to our knowledge, no one has ever reported previously.

#### Delayed wound-healing time

**Figure 5:**
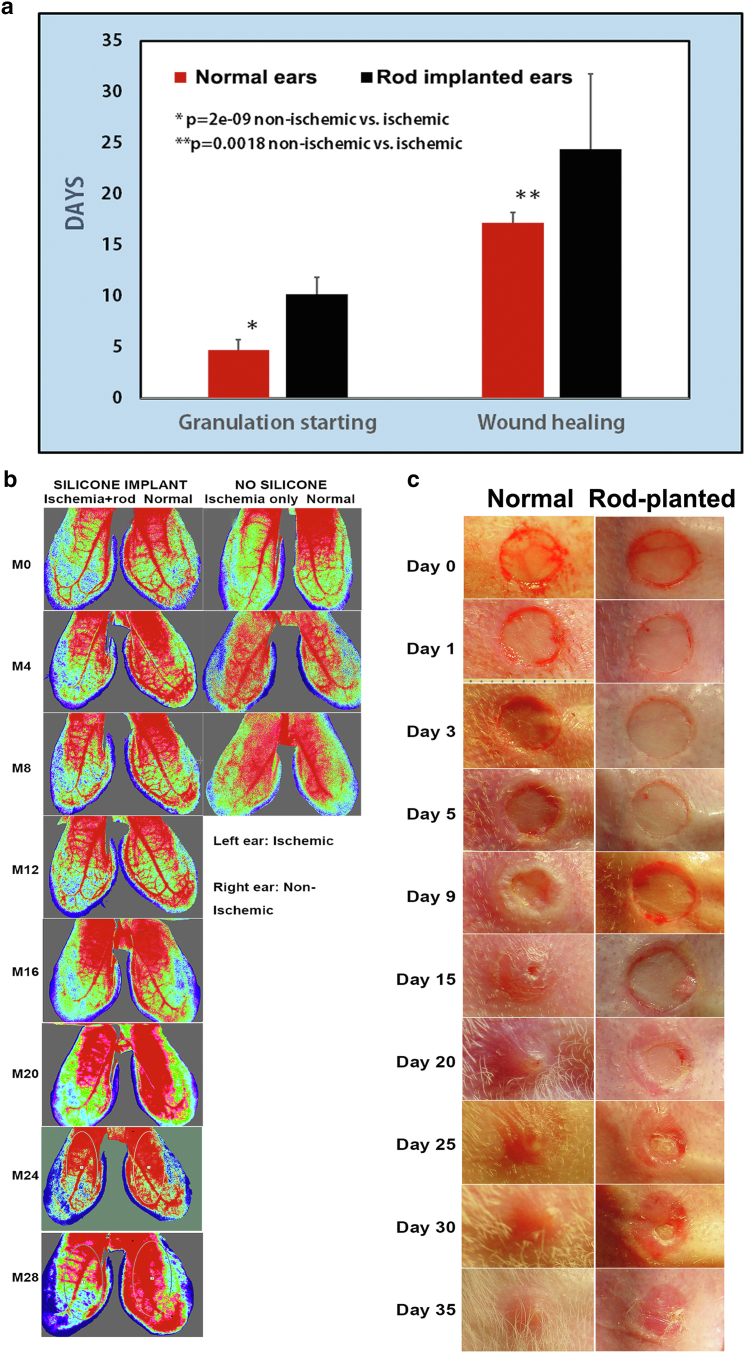
**Granulation tissue and wound-healing comparisons.** Shown are differences in granulation tissue initiation time and wound-healing comparison between normal ears and ischemic ears. **(a)** Comparison of granulation tissue initiation time and wound-healing durations between ischemic and nonischemic groups (8 animals, 32 wounds in each group). On the nonischemic wounds, granulation started to appear at 4.75 ± 1.23 days (mean ± SEM) compared with that in ischemic ears where granulation started at day 10.18 ± 1.68 (*P* < .0001). Healing was noted at day 17.20 ± 4.38 in nonischemic ears compared with 24.43 ± 7.38 days for ischemic ears (*P* = .0018). Student *t-*test was used as described in the statistical analysis section in the text. **(**b**)** An example of ear tissue perfusion differences between the ischemic ears with and without silicone rod implant. The difference in the ears without silicone rod implant, the difference disappears after 1 month, but the difference was maintained at 28 months in the silicone rod–implanted ear. **(c)** An example of the wound granulation and healing time differences between the normal ears and the ears with silicone rod implant. On the normal ears, the wound is essentially healed at day 20. On the silicone rod–implanted ears, the wound is even not totally healed at day 35.

We generated ear skin wounds on the ventral side of the ears of 8 rabbits (64 wounds). Both the granulation tissue starting and the wound-healing times were much longer in the ischemic wounds with silicone rod implant than in the nonischemic wounds (Figure 5a and b). The example provided in Figure 5c shows that the wounds on the silicone rod–implanted ischemic ear were still not totally healed at postoperative day 35. To our knowledge, such a large healing difference has not been reported in any other model.

#### Histology study

**Figure 6:**
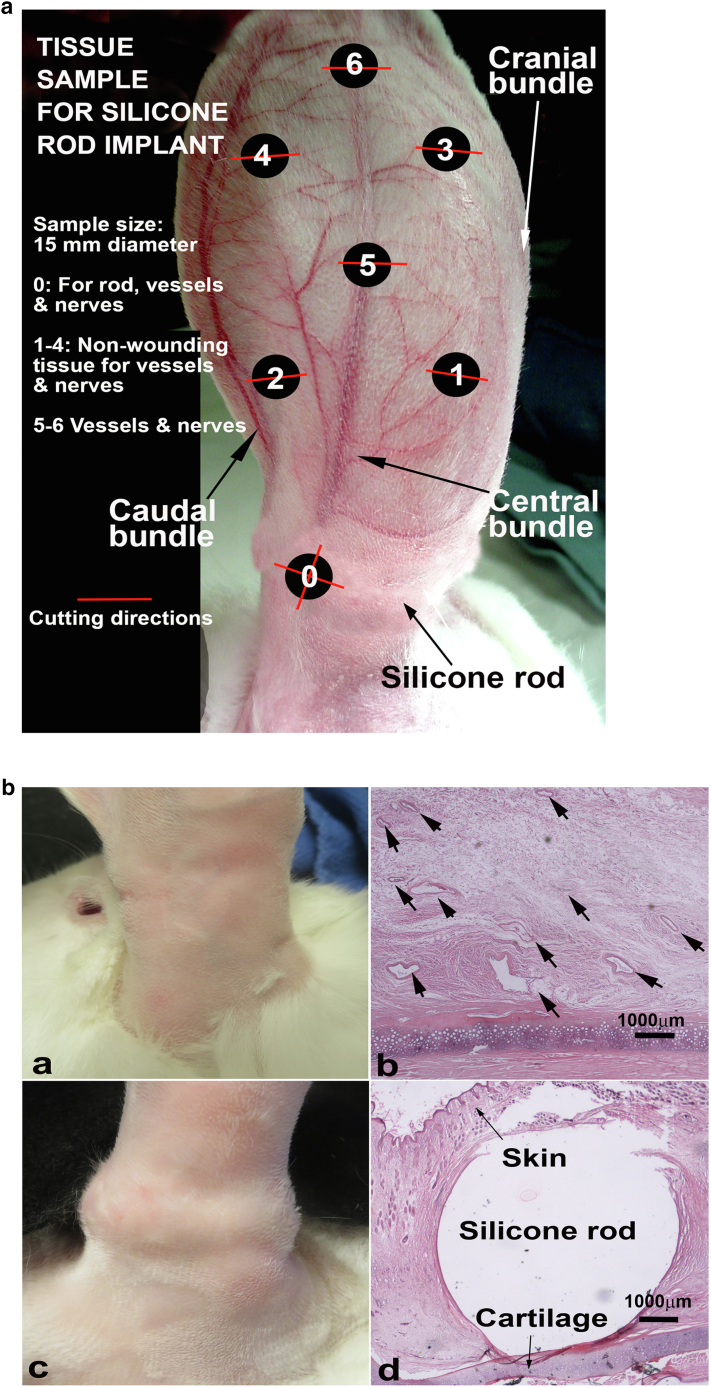
**Tissue sample and histology differences.** Ear tissue sample sites and the histological differences between ear ischemia with and without silicone rod implant are shown. **(a)** Ear tissue sample sites. The samples were taken at difference sites on each ear. The number 0 is at the ear base where silicone rod is implanted. The numbers 1–4 are the ear tissue without major blood vessels, whereas 5 and 6 are taken from the sites where the central bundle is located. **(b)** Ear base outlook (for **a** and **c**) and H&E staining of samples 6 months after surgery (for **b** and **d**) (from site 0 in **a** above). In the ear base without silicone rod implant (for **a** and **b**), multiple blood vessels have been regenerated (for **b**, arrows). In the silicone rod implanted ear base (for **c** and **d**), the silicone rod has taken most of the space, leaving very little space for possible vessel and nerve regeneration (for **d**, arrows). Animals used = 27. Bar = 1000 mm.

Histologically, the subcutaneous tissue at the ear base was totally dissected during surgery in the regular ischemic ear, but collateral vessels were eventually re-established after 1 month. The implantation of silicone rods in the extended ischemic group blocked most of the subcutaneous tissues, and ear perfusion was reduced for a much longer time. We took tissue samples from different locations of the silicone rod–implanted ear to compare perfusion (Figure 6a), whereas the regular ischemic ear has already generated plenty of collateral vessels (Figure 6a and b). This low perfusion is mainly due to the lack of collateral formation because the implanted silicone rod occupied most of the tunnel space, resulting in very little collateral vessel formation (Figure 6c and d).

#### Immunohistochemistry

**Figure 7:**
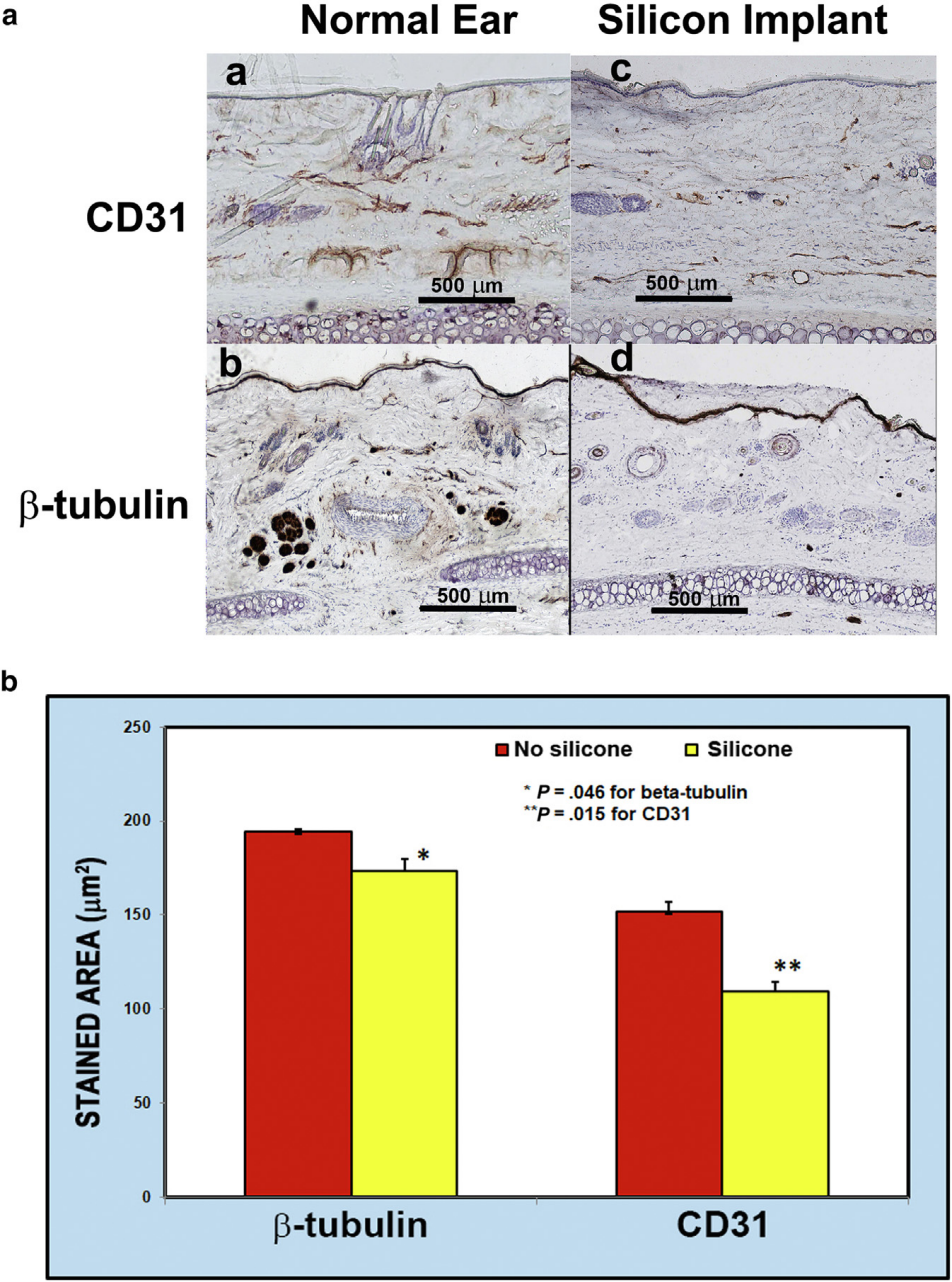
**Immunohistochemistry.** Immunohistochemistry analyses and differences between the wounds with and without silicone rod implant are presented. **(a)** Stains of CD31 and ß-tubulin in ear tissues 6 months after surgery. **a–d** are taken from the sites of 1–4 in Figure 6a. **a** and **c** show CD31 staining in the ear tissue 6 months postoperative (**c**) with and (**a**) without silicone implant. **b** and **d** show b-tubulin staining in ear tissue 6 months postoperative (**d**) with or (**b**) without silicone implant. The yellow–brown color shows positive of these 2 antibodies. Bar = 500 mm. All experiments were carried out in triplicates. **(b)** This shows mean plus SD of ß-tubulin and CD31-stained areas (μm^2^) in nonsilicone (normal) ears compared with that in silicone rod–implanted ears (ischemic), which were analyzed using the Student *t*-test. Both stains were performed in triplicates. The results have shown significant differences in the stained area of ß-tubulin (190 ± 2 μm^2^) and CD31 (150 ± 4 μm^2^) in nonsilicone ears compared with that in silicone rod–implanted ears (170 ± 5 μm^2^ and 110 ± 4 μm^2^, respectively).

Our immunohistochemistry results showed that the silicone rod–implanted ears had lower expression of CD31 and β-tubulin, and this reduced expression lasted for 6 months after surgery (Figure 7a and b). This finding supports our theory that silicone rod implants can prevent vessel and nerve regeneration for a much longer time.

### Possible complications and other precautions

Because the ischemia is more severe and much longer than that produced by the regular ischemic ear procedure, several problems may result, and we observed them in the early days of the procedure development. However, these were not caused by the technique itself and could be avoided by careful selection of material and appropriate manipulations. These include the following:

#### Tissue edema in the ischemic ear

The best approach for the rod implantation procedure is to keep both the central and caudal veins intact, and this is especially true for the central vein. In a few cases where the central veins were damaged, severe postoperative edema developed. When no contusion occurred on the skin, the edema would disappear in approximately a week. However, if skin contusions were evident on the ear, healing was much slower, and this could affect the study of normal wound healing. Keeping the major vein patent prevented this complication.

#### Silicone rod end separation, pinching, and perforation

**Figure 8:**
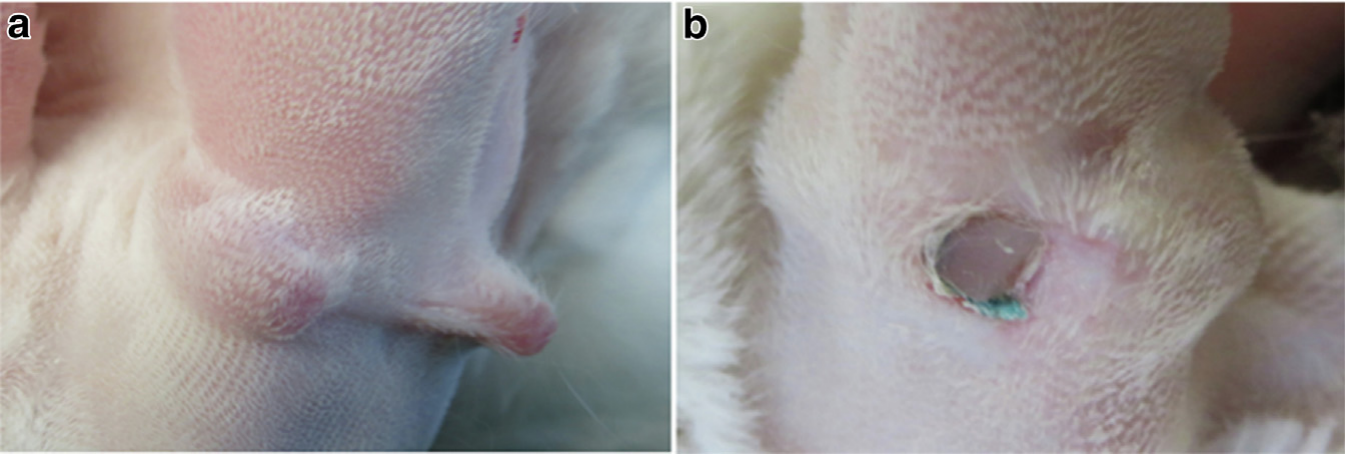
**Possible complications.** The possible complications of the silicone rod implant are discussed. Silicone rod separation may occur at the end where the 2 sides sutured together. The silicone rod separation causes (**a**) ear base tissue pinch and (**b**) possible perforation. Because the silicone rod has a very good tissue compatibility, removal of the exposed silicone rod can eliminate any infection (as seen in **b**). Using softer silicone rings (instead of rod) can essentially eliminate this complication.

These events occurred when the ends of the rod became separated owing to strain on the rod. This separation depends on the physical characteristics of the silicone rods, including their hardness and shape. When we used a straight silicone rod (Sil-Pro), the 2 ends were sutured together at least twice, but some of them still broke after a few weeks. Sil-Pro has a hardness of Shore A 50, which is relatively firm, resulting in skin pinching or perforation (Figure 8a and b). The use of a silicone O-ring (Atlantic Rubber) with the same hardness almost completely eliminated the chance of breaking off because it put much less strain on the ends. Using a rod from chemical processing by ourselves, such as the OOMOO (Smooth-on), allowed the production of a much softer silicone rod with Shore A 20–30 in the durometer; this had much less chance of breaking the skin. Because our selected silicone rod or ring has no toxicity with good tissue bioaccumulation, we often just cut the exposed portion and the skin healed around the rod, resulting in no trouble at all for many months (Figure 8b).

#### Skin incision

Two approaches were used for the original rabbit ear ischemic procedure. The original was a circumferential incision to make the tunnel (Ahn and Mustoe, 1990). Our group modified the technique using a minimally invasive approach resulting in 3 small skin incisions (Chien, 2007; Chien and Wilhelmi, 2012). The technique reduced skin damage significantly. The current surgery requires implantation of the silicone rod under the incision, which will put tremendous pressure on the ear base skin. A circumferential skin incision should be avoided because the silicone rod is immediately underneath the skin incision and experiences significant pressure that could give rise to complications. Our suggestion is to only use a minimally invasive approach for the silicone rod implant to reduce the possibility of incision breakage or separation.

#### Silicone rod movement

Notches are made over the silicone rods to avoid any pressure on the 2 vascular branches because any movement of the silicone rod can damage the vessels, resulting in severe edema and/or necrosis. For this reason, the silicone rod needs to be fixed onto the cartilage. As described in Materials and Methods, the perichondrium needs to be preserved, and at least 2 sutures are used to fix the silicone rod to the perichondrium and cartilage. We have not seen a single movement of the implanted rods.

## Discussion

In past decades, more than 1000 in vitro and in vivo wound models have been developed, but none of them has successfully recapitulated the basic pathophysiology of 3 major types of human chronic wounds—venous ulcers, diabetic foot ulcers, and pressure ulcers (Medina et al, 2005; Nunan et al, 2014)—in terms of their clinical features. Reasons for this failure are as follows: (i) all models are for acute, not chronic, wounds; (ii) most models do not also include ischemia, even the few that incorporate ischemia have too short an ischemic time; (iii) most models are created in young animals, but the physiologic response to wounding is disrupted in the elderly; and (iv) many models are created in mice or rats, but murine skin undergoes myofibroblast-mediated contraction owing in part to an extensive subcutaneous striated muscle layer called the panniculus carnosus, which is mostly absent in humans (Grada et al, 2018). The difference between murine and human genomes is also significant unless the trauma is sufficiently severe to trigger a "genomic storm" (Gentile et al, 2014; Xiao et al, 2011). The cumulative effect is that topical agents that improve wound healing in the young typically fail to work in the seniors. Similarly, small-scale clinical trials of GFs, such as basic fibroblast GF, have shown effectiveness in nonischemic wounds, but their effects disappear in hypoxic dermal ulcer models (Lindblad, 2006). Without a suitable animal model that recapitulates the precise environment of a chronic wound, an understanding of the mechanisms that act to produce this type of wound or that delay healing could remain out of reach (Salcido et al, 2007).

As stated earlier, the most critical pathophysiology of nonhealing chronic wounds is associated with a deficient blood supply. This principle applies to all major chronic wounds, such as diabetic ulcers (Levigne et al, 2013), venous ulcers (Wollina et al, 2006), and pressure ulcers (Liao et al, 2013), as well as wounds with arterial insufficiency (Bauer et al, 2005). All surgeons recognize that wounds in ischemic tissues heal poorly or not at all, whereas wounds in highly vascular tissues heal rapidly (Niinikoski, 2002). Furthermore, once an ulcer has developed, the blood supply required for wound healing increases many folds compared with that required for adequate nutrition of the same volume of intact skin (Lepantalo et al, 2000).

For many chronic wounds, ischemia may not be the initiating factor for nonhealing because most ulcers start from a combination of repeated trauma, neuropathy, pressure loading, and infection (Fang and Galiano, 2008; Medina et al, 2005). However, tissue ischemia is the main cause of delayed healing. Simply increasing the wound oxygen supply with hyperbaric oxygen therapy has not shown consistent results (Berendt, 2006); this is because a lack of oxygen is only 1 part of ischemic pathophysiology—the most critical consequence of ischemia is a decrease in cellular energy supply (Im and Hoopes, 1970; Smith et al, 1999). Energy is required in every aspect of the wound-healing process (Hunt and Pai, 1972; Wang et al, 1990).

The most popular procedure for producing an ischemic model is to form a U-shaped cutaneous flap on the back of rats, as first reported by McFarlane et al (1965) more than half a century ago and subsequently followed by numerous modifications (McFarlane et al, 1965; Dunn and Mancoll, 1992; Schaffer et al, 2002). The major problem with this model is the short ischemia period because new vascular channels are present around the entire wound margin and develop from the recipient bed within 2–3 days (Nakajima, 1978). Consequently, blood perfusion increases in a linear fashion and reaches normal levels at postoperative days 14–16 (Sisco and Mustoe, 2003).

A relatively longer duration of ischemia has only been achieved with a rabbit ear model by ligating 2 of the 3 arteries to create ischemia. Tissue partial pressure of oxygen was significantly decreased postoperatively, and the decrease lasted for 28 days (Ahn and Mustoe, 1990; Sisco and Mustoe, 2003). However, this ischemic time is still too short for chronic wounds.

Rabbits are recognized as a more reliable disease model for the development of therapeutics and the elucidation of cellular and molecular mechanisms underlying many human diseases (Esteves et al, 2018; Graur et al, 1996). Rabbits are also closer to humans in evolutionary trees than many other animals currently used as models (Gentile et al, 2014; Zindle et al, 2021). Using rabbits for wound study is a suitable approach. The current ischemic wound model, after a long-time exploration, has finally achieved stable and maintained a much longer time of ischemia suitable for wound-care studies. We are working on many more animal tests, but the potential contribution to wound care will be significant.

To our knowledge, this is a previously unreported animal model showing extremely long-term ischemia with nerve damage suitable for wound study. The model can be easily combined with other pathological changes such as diabetes, reduced blood supply, and aging. Our results have shown no severe complications even with extended wound-healing time when done carefully.

## Materials and Methods

The technique can produce the longest reported ischemic time—from several months to over 2 years. It can also be combined with diabetes, aging, and infection to simulate the features of different human chronic wounds.

This approach involves blocking collateral formation with a solid material, thereby simultaneously preventing both nerve and vessel recovery.

### Animals

A total of 55 young adult New Zealand White rabbits were used (both male and female, aged 6–12 months, body weight = 2.5–3.5 kg, Charles River Laboratories). Twenty were used for early exploration of the technique and for regular ischemic ear model creation, whereas the other 35 were used for the extended ischemic model with silicone rod implantation. Among these 35 rabbits, 8 were diabetic, which was generated using alloxan as reported previously by our group (Wang et al, 2010).

### Selection of vascular and nerve blockage materials

We tested multiple silicone rod (or O-ring) materials and finally selected a ready-made silicone rod (Sil-Pro, Trelleborg Healthcare and Medical) and a silicone O-ring (Atlantic Rubber). All materials were checked first in cell culture to ascertain the absence of toxicity followed by in vivo implants observed for a month without tissue reaction and unusual findings in histologic examination.

### Induction and management of diabetes mellitus

This study was approved by the Institutional Animal Care and Use Committee of the University of Louisville. Diabetes was in 8 young New Zealand white rabbits (aged 6–12 months, body weight = 2.5–3.5 kg). Diabetes mellitus was generated by injection of alloxan (Sigma Aldrich) as reported earlier (Wang et al, 2010). Briefly, alloxan (100 mg/kg) was dissolved in normal saline to a concentration of 5% (w/v). This 5% alloxan was administered intravenously through the marginal ear vein over a period of 1 minute to the lightly anesthetized rabbits (25 mg/kg ketamine hydrochloride and 2.5 mg/kg xylazine, intramuscular). A total of 10 ml glucose solution (5% w/v) was administered subcutaneously 4, 8, and 12 hours after alloxan injection. Glucose (20%) was provided as drink for 1–2 days to offset transient hypoglycemia. Hyperglycemia was observed to develop after 48 hours of alloxan injection and was stabilized after a week (Wang et al, 2010). Owing to the injection of alloxan, the pancreatic β cells were severely destroyed, and hence daily insulin injection was required (Wang et al, 2010). Blood glucose concentration was measured using a blood glucose meter (LifeSpan) once/twice daily for the first 4 weeks and once every week thereafter. For blood glucose level >350 mg/dl, 1–4 U/kg regular insulin (Novolin, Novo Nordisk Pharmaceuticals) was injected subcutaneously once or twice a day (depending on blood glucose levels). All the 8 animals were maintained diabetic for more than 6 months by recording their blood glucose levels and body weight every week. Blood and urine samples from the rabbits with diabetes were collected for biochemical analysis weekly for the initial 2 months and later monthly until the end of experiment (Wang et al, 2010).

### Surgical procedure

The technique is an extension of the minimally invasive approach developed by our group (Chien, 2007; Chien and Wilhelmi, 2012) and was called “UofL Model.” Animal study was conducted in accordance with the National Institutes of Health Guidelines for the Care and Use of Animals in Research, and the protocols (IACUC 14059-20812) were approved by the Institutional Animal Care and Use Committee of the University of Louisville, an American Association for Accreditation of Laboratory Animal Care–accredited program.

**Figure 9:**
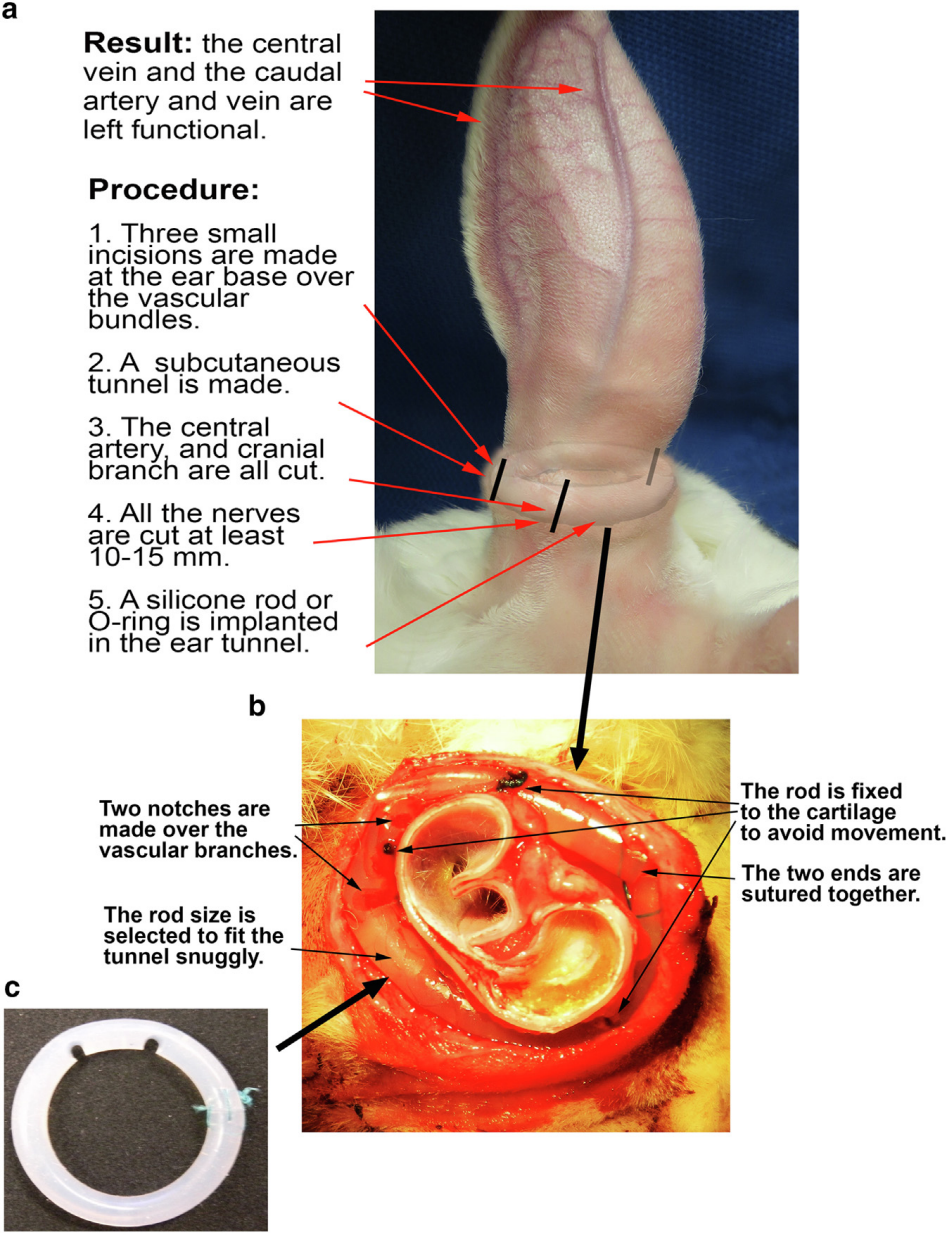
**Photographic summary.** The photographic summary of the silicone rod implant procedure is presented. **(a)** An overview of the surgical procedure to implant a silicone rod in the ear base. The description explains the steps we used to achieve the result. **(b)** A section of the ear base with the silicone rod implant. After the tunnel is made, a silicone rod is placed in the tunnel, and the 2 ends are sutured together. The silicone rod fills most of the space in the tunnel, and this can permanently block blood vessel and nerve regenerations. **(c)** Silicone rod in vitro. Two notches are made on the vessel sites to prevent the silicone rod from compressing the vessels when in place.

The rabbit ear is supplied by 3 vascular pedicles. The central bundle is the largest, the cranial is smaller, and the caudal branch is the smallest. These vessels are easily seen through the skin after the ears are shaved (Figure 9a). The skin on the ventral side of the ear does not carry the blood supply to the distal portion. The 3 vessel bundles on the dorsal ear not only supply blood to the outside ear skin but also to the ventral skin by numerous penetrating branches through the cartilage. In some rabbits, there is a very small subcutaneous vessel on the ventral side of the ear, which supplies blood to no more than one third of the ventral skin. In an open circumferential skin incision, there is essentially no significant bleeding from the ventral side. As such, the inside incision can be avoided. On the basis of these observations, we have used the minimally invasive approach for creating the ischemic ear model for wound research. The procedure is to make 3 small vertical skin incisions on the 3 vascular pedicles at the ear base to ligate and divide the arteries. A subcutaneous circumferential tunnel is made through the skin incisions to disrupt the subcutaneous tissues, muscles, nerves, and other vessels. No skin incision is made through the ear rim to the ventral side. As such, skin continuation is preserved (Chien, 2007).

Under general anesthesia (50 mg/kg ketamine + 5 mg/kg xylazine, intramuscular) and with vital organ functions monitored, both ears were shaved and prepared aseptically with a surgical scrub and draped. The procedure was as follows:

1. One ear was rendered ischemic, whereas the other ear served as a paired nonischemic control. Using a #15 blade, 3 small vertical incisions (1–1.5 cm) were made on the vascular pedicles about 1 cm distal to the base of the ear.
2. Three vascular pedicles were identified. The central artery was ligated with 4–0 prolene, divided, and the accompanying nerve was cut as well. The cranial bundle was ligated and divided. The caudal bundle was preserved.
3. A circumferential subcutaneous tunnel was made at the base of the ear through the 3 incisions. This was accomplished by sharp dissections with a knife and scissors. All the subcutaneous tissues, muscles, nerves, and small vascular branches were discontinued. The small vasculatures underneath the dermis were totally divided.
4. Instead of dissecting the perichondrium down to the cartilage, the perichondrium was preserved to facilitate suturing to anchor the silicone rod.
5. A selected silicone rod (or O-ring) was threaded through the tunnel to completely fill the tunnel and sutured end to end (Figure 9b). The diameter of the silicone rod or ring ranged from 3 to 10 mm, with a 5-mm diameter used in most rabbits.
6. At the sites where the central and the caudal bundles were located, 2 notches were made on the silicone rod to avoid compression of the vessels (Figure 9c).
7. To prevent the movement of the silicone rod, 2–3 stitches were made to fix the rod to the ear base. The sutures passed through the perichondrium, cartilage, and the silicone rod but did not perforate the cartilage (Figure 9b).
8. The silicone rod length was controlled to fit the ear base snuggly to block collateral formation but without substantial expansion of the skin.
9. The 2 ends of the silicone rod were sutured together with at least 2 stitches to make the ends closed as smoothly as possible to avoid any skin pinching.
10. Before the skin was closed, the operation area was checked carefully to confirm that the remaining vessels were not obstructed, no portion of the ear skin was pinched, and no bleeding occurred.
11. The procedure interrupted most subcutaneous vascular branches so that they would not become collaterals. Bleeding was minimal and easily controlled by gauze compression without using electrocautery. The skin inside the ear does not carry much blood supply and thus was not incised. The 3 skin incisions were closed using 4-0 or 5-0 sutures and covered with sterile gauze. This procedure resulted in 3 small skin incisions and incurred much less damage.
12. For wound studies, 4 circular full-thickness wounds were created on the ventral side of each ear with a 5–6-mm stainless steel punch (Figure 10a). The distance between the wounds was 20–30 mm. The skin inside the punch wound was removed from the cartilage. The perichondrium was also removed. The base on which granulation took place was the cartilage, but the cartilage was not perforated.

**Figure 10:**
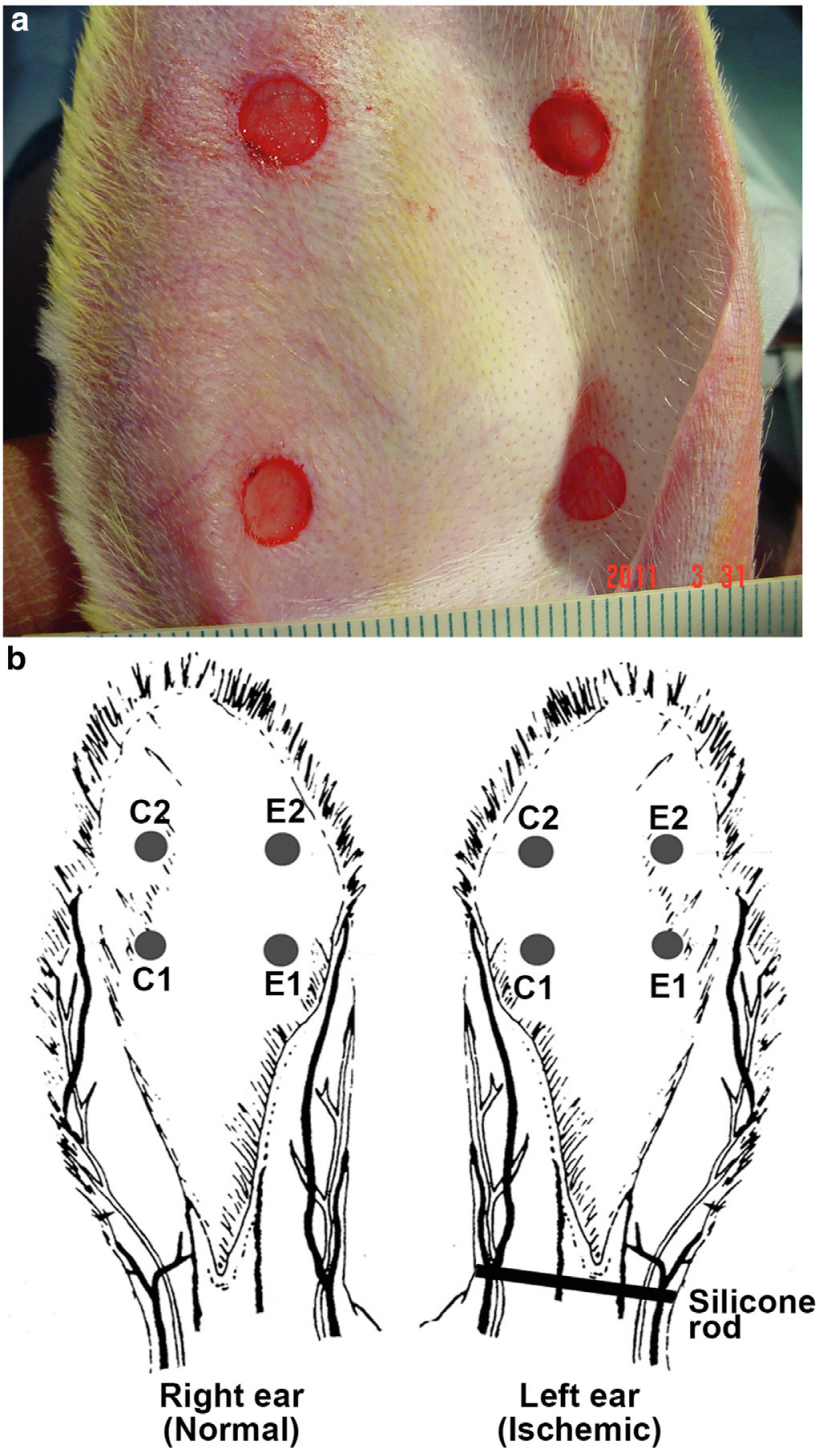
**Photograph illustrations.** Shown are photograph and illustration of rabbit ear ischemic model generation and wound creation. **(a)** Photograph of 4 wounds made on the ventral side of the ear. The distance between each wound is 20–30 mm. **(b)** Illustration of wound creation on the ears and their dressing sites. The 2 wounds on one side are used for testing experimental drugs (E1 and E2), and the 2 wounds on the other side are used for control dressings (C1 and C2). The left ear is made to show strong ischemia with vessel and nerve reduction plus silicone rod implant to reduce the possibility of vessel and nerve regeneration. The right ear is used as the normal control.
13. After the creation of wounds on both the ears, a small piece of gauze or cotton was applied to the wounds, and normal saline or other testing solutions were used on each side as paired comparison (Figure 10b). An occlusive dressing (TegaDerm, 3M) was used to cover the wound site. This prevented the wounds from becoming desiccated.

### Postoperative managements

Meloxicam, 0.1 mg/kg, was given by mouth once a day for 3 days to reduce pain. The ears were checked daily for physical appearance, and tissue perfusion was measured daily for the first 2 weeks and every week for the remainder of the experimental period.

The created wounds were managed every day, and dressing changes were made daily. The old dressings were removed, the wounds were cleaned carefully, and photographs were taken daily with scales for wound and granulation size measurements and comparisons. The wounds were covered with TegaDerm until healed or until the animals were killed. Wound healing and ischemic times were measured in all animals.

### Histology and immunohistochemistry

Rabbits were killed on different days after surgery. Wound samples were taken with a circular punch from different areas of the ear with silicone rod implant and compared with a circular punch from the normal ear. Tissue samples were fixed, processed, sliced to 5-μm thickness, and stained, as reported earlier (Sarojini et al, 2017). Briefly, tissue samples were fixed in 4% buffered formaldehyde and then paraffin embedded for histologic and immunohistochemical studies. A total of 5-μm slices were cut from the paraffin blocks and mounted on Superfrost Plus microscopic slides (Thermo Fisher Scientific). These serial sections were further stained by H&E, beta III-tubulin, and CD31 antibodies (5-μm thickness) on the slide. For immunostaining, the paraffin sections were deparaffinized in xylene and rehydrated through a series of graded alcohol, ending with Tris buffered saline containing Tween 20 (Thermo Fisher Scientific). Antigen retrieval was done at 100 °C in immunohistochemical antigen retrieval solution High/Low pH (Ebioscience) for 1 minute. The endogenous peroxidase activity was quenched by incubation with 0.3% hydrogen peroxide (Thermo Fisher Scientific), and nonspecific antibody binding was blocked with RTU Serum-free Protein Block (Dako) for 20 minutes. The slides were then incubated with primary antibody at 37 ^o^C for 1 hour. The presence of neurons was detected using mouse monoclonal anti-beta III tubulin antibody (Abcam) immunostaining. The differences in vascularization in ischemic and nonischemic samples were detected using mouse monoclonal anti-CD31 (Abcam) antibodies.

These were subsequently incubated with RTU EnVision+ HRP Labeled Polymer anti-mouse second antibody (Dako) at 37 °C for 30 minutes. The sections were rinsed after each reaction. Finally, the immunoreaction products were visualized with a solution of DAB+ (Dako). The histological samples were visualized and imaged using a Nikon Eclipse Ti fluorescence microscope (Nikon Instruments). Zeiss Axio Imaging software was used for morphological analysis of the images.

### Statistical analyses

Wounds and all physiologic results were compared among the normal, regular ischemic, and extended ischemic tissues (with silicone rod implant) using commercial software (Statistical Package for the Social Sciences 26, 2018 by IBM) according to ischemic time, histological and immunohistochemical differences, skin temperature, and wound healing times. The 2 ears in rabbits have provided the ideal paired ears—ischemic-left, nonischemic (normal)-right—with good comparison. We first used the null hypothesis to examine whether the 3 groups (normal, ischemia without silicone rod implant, and the group with silicone rod implant) and found this hypothesis rejected because there were significant differences in 3 groups in all parameters related to ischemia and wound healing. After that, the normal distribution of data was assessed using the Shapiro–Wilks test for normality. Owing to substantial disparities between ischemic and normal ear perfusions, our study clearly demonstrates the normality and homogeneity of variances. Student’s *t*-test is was to compare the means between 2 groups, whereas ANOVA was used to compare 3 or more groups. All values were presented as mean and SEM or mean and SD. When a *P* < .05, it was considered statistically significant.

## Ethics Statement

The animal study was conducted in accordance with the National Institutes of Health Guidelines for the Care and Use of Animals in Research, and the protocols (IACUC 14059-20812) were approved by the Institutional Animal Care and Use Committee of the University of Louisville, an American Association for Accreditation of Laboratory Animal Care–accredited institution.

## Data Availability Statement

No large datasets were generated or analyzed during this study. Minimal datasets necessary to interpret and/or replicate data in this paper are available upon request to the corresponding author.

## Conflict of Interest

The authors state no conflict of interest.

## Declaration of Generative Artificial Intelligence (AI) or Large Language Models (LLMs)

The author(s) did not use AI/LLM in any part of the research process and/or manuscript preparation.
